# A Silent Mimic: Biopsy-Proven Focal Nodular Hyperplasia Masquerading as Chronic Intrahepatic Biliary Obstruction

**DOI:** 10.14740/jmc5364

**Published:** 2026-09-04

**Authors:** Imroz Apon, Ahmed Shehadah, Behrawar Ahmed, Usama Sakhawat, Ali Marhaba, Chibuike Enwereuzo

**Affiliations:** a Department of Internal Medicine, United Health Services, Johnson City, NY, USA; b Department of Gastroenterology, United Health Services, Johnson City, NY, USA; c Department of Pathology, United Health Services, Johnson City, NY, USA

**Keywords:** Focal nodular hyperplasia, Biliary obstruction, Liver mass, Cholestatic liver injury, Liver biopsy, Hepatic adenoma

## Abstract

Focal nodular hyperplasia (FNH) is a benign hepatic lesion that is typically asymptomatic and most often found incidentally. Biliary obstruction caused by FNH is rare and can create diagnostic uncertainty, particularly when imaging can mimic vascular lesions, hepatocellular adenoma, bile duct adenoma, or congenital hepatobiliary abnormalities. We present the case of a 38-year-old woman with hypertension who was noted to have persistently abnormal liver enzymes on routine outpatient blood tests. She denied symptoms such as jaundice, pruritus, pale stools, dark urine, weight loss, nausea, vomiting, or abdominal pain. Laboratory tests showed a chronic cholestatic-predominant pattern with elevated alkaline phosphatase, mildly elevated aspartate aminotransferase (AST) and alanine aminotransferase (ALT), fluctuating hyperbilirubinemia, and markedly elevated gamma-glutamyl transferase (GGT). Ultrasound revealed intrahepatic biliary ductal dilatation and indeterminate cystic areas within the liver. Computed tomography revealed chronic biliary ductal dilatation, hepatic parenchymal deformity, a mass-like hypertrophied hepatic segment, and a central arterial-enhancing structure initially concerning for aneurysm or pseudoaneurysm. Magnetic resonance imaging (MRI) and magnetic resonance cholangiopancreatography (MRCP) demonstrated marked intrahepatic biliary dilatation, an arterially enhancing lesion suspicious for atypical FNH, and a second exophytic lesion causing mass effect on the biliary tree. Differentials included atypical adenoma, intrahepatic bile duct adenoma, vascular lesion, or congenital anomaly. Because serial imaging remained indeterminate, percutaneous liver biopsy was performed. Histopathology showed preserved lobular architecture, intermittent thin fibrous septa, focal sinusoidal dilatation, ductular reaction, and map-like glutamine synthetase staining with membranous beta-catenin staining, supporting the diagnosis of FNH. This case highlights an atypical presentation of FNH causing chronic intrahepatic biliary obstruction and emphasizes the importance of integrating imaging, multidisciplinary review, and histopathology when benign hepatic lesions present atypically and influence management decisions.

## Introduction

Focal nodular hyperplasia (FNH) is one of the most common benign hepatic lesions typically encountered in women of reproductive age. It is generally regarded as a hyperplastic response to localized vascular abnormalities rather than a true neoplasm. Most cases are asymptomatic and discovered incidentally. In many patients, magnetic resonance imaging (MRI), especially with hepatobiliary-specific contrast agents, can establish the diagnosis noninvasively; however, atypical lesions may remain difficult to distinguish from hepatocellular adenomas because of overlapping imaging features [1–5]. Benign lesions such as FNH and hemangiomas are usually found on segment four on Couinaud’s classification. Our case involved lesions in both segment four and segment 6 [6].

Biliary obstruction due to FNH is rare but may present with a chronic cholestatic pattern of liver enzyme abnormalities and progressive biliary ductal dilatation. In such cases, the differential based on radiological findings may broaden substantially, especially when lesions are centrally located or associated with hepatic remodeling and mass effect on the biliary tree [7, 8].

We present a case of biopsy-proven FNH in a 38-year-old woman who developed chronic intrahepatic biliary obstruction with persistent abnormal liver biochemical tests and complex imaging findings initially concerning for vascular lesion, adenoma, bile duct adenoma, or congenital hepatobiliary disease.

## Case Report

A 38-year-old woman with a past medical history significant for hypertension was found to have abnormal liver enzymes during routine laboratory testing by her primary care provider. She had no known personal or family history of liver disease, biliary disease, autoimmune disease, or inflammatory bowel disease. She had never undergone cholecystectomy or any prior abdominal surgery and denied any history of abdominal trauma. She has no prior use of oral contraceptive pills.

Approximately 1 year before tissue diagnosis, initial laboratory testing showed an incidental finding of elevated liver enzymes with aspartate aminotransferase (AST) 80 U/L, alanine aminotransferase (ALT) 60 U/L, alkaline phosphatase 292 U/L, and total bilirubin 1.1 mg/dL. This was attributed to acetaminophen use, as she reported that she had used approximately three bottles of acetaminophen over the prior 3 months because of back pain and headaches. She denied alcohol use, smoking, and recreational drug use. Repeat hepatic function testing continued to show persistent abnormalities, with AST 90 U/L, ALT 64 U/L, alkaline phosphatase 181 U/L, and total bilirubin 1.2 mg/dL. Over time, the patient continued to demonstrate chronic mild transaminitis, persistent elevation in alkaline phosphatase, and fluctuating bilirubin elevation. Additional laboratory data showed elevated gamma-glutamyl transferase 476 U/L, albumin ranging from 3.9 to 4.8 g/dL, prothrombin time 12.2 to 15.0 s, international normalized ratio 1.04 to 1.18, and activated partial thromboplastin time 30.9 to 38 s. Viral hepatitis workup was negative, including hepatitis A immunoglobulin (Ig)M antibody, hepatitis B core IgM antibody, hepatitis B surface antigen, and hepatitis C antibody. Total immunoglobulin G was elevated at 2,016 mg/dL, with elevated IgG subclass 2 at 1,056 mg/dL, while IgG subclass 4 was normal. No additional autoimmune serologies were obtained.

Despite these abnormalities, the patient remained asymptomatic. She denied scleral icterus, jaundice, pruritus, pale stools, dark urine, postprandial abdominal pain, nausea, vomiting, and weight loss. Her appetite remained normal, and her weight was stable.

**Figure 1:**
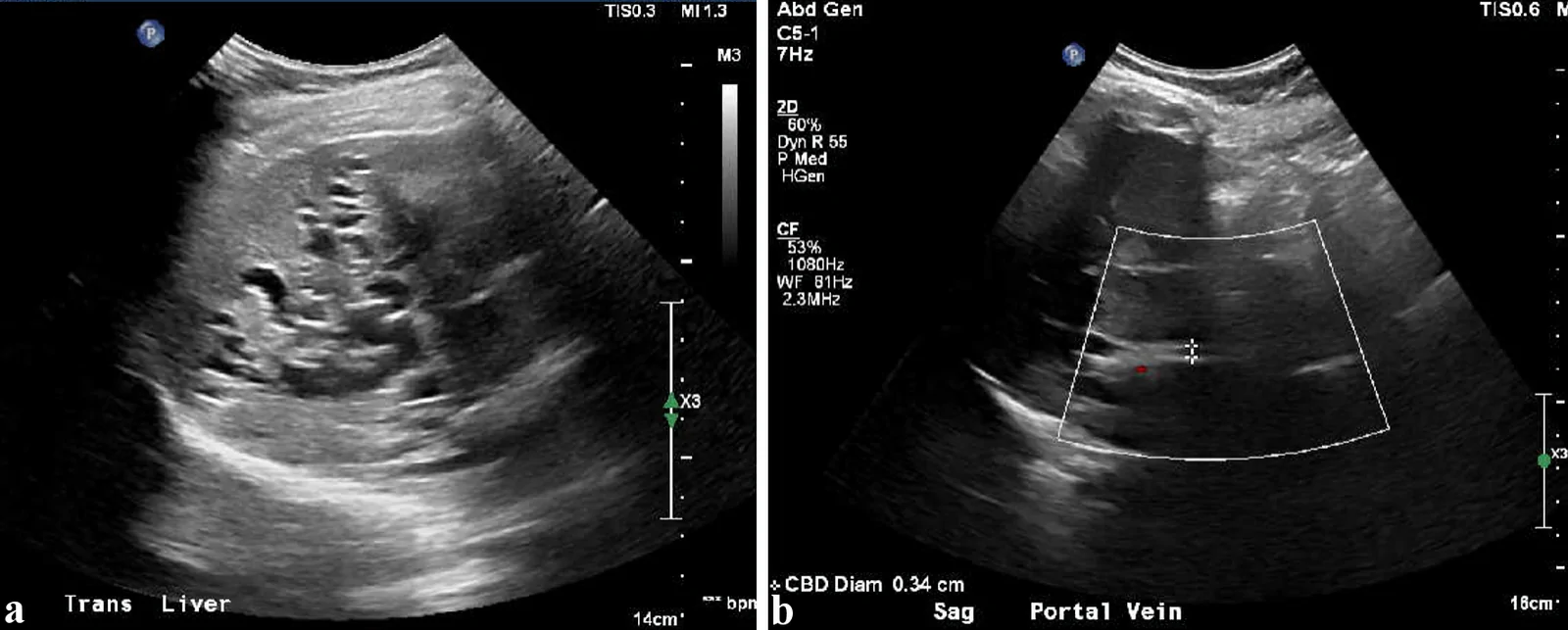
Ultrasound complete abdomen, showing (a) multiple cystic dilations in the liver and (b) common bile duct measuring 0.34 cm.

A right upper quadrant ultrasound demonstrated numerous dilated structures within the liver that were difficult to distinguish between cysts and biliary ductal dilatation (Fig. 1a). The common bile duct measured 3.4 mm (Fig. 1b). The portal vein was patent with normal hepatopetal flow.

**Figure 2:**
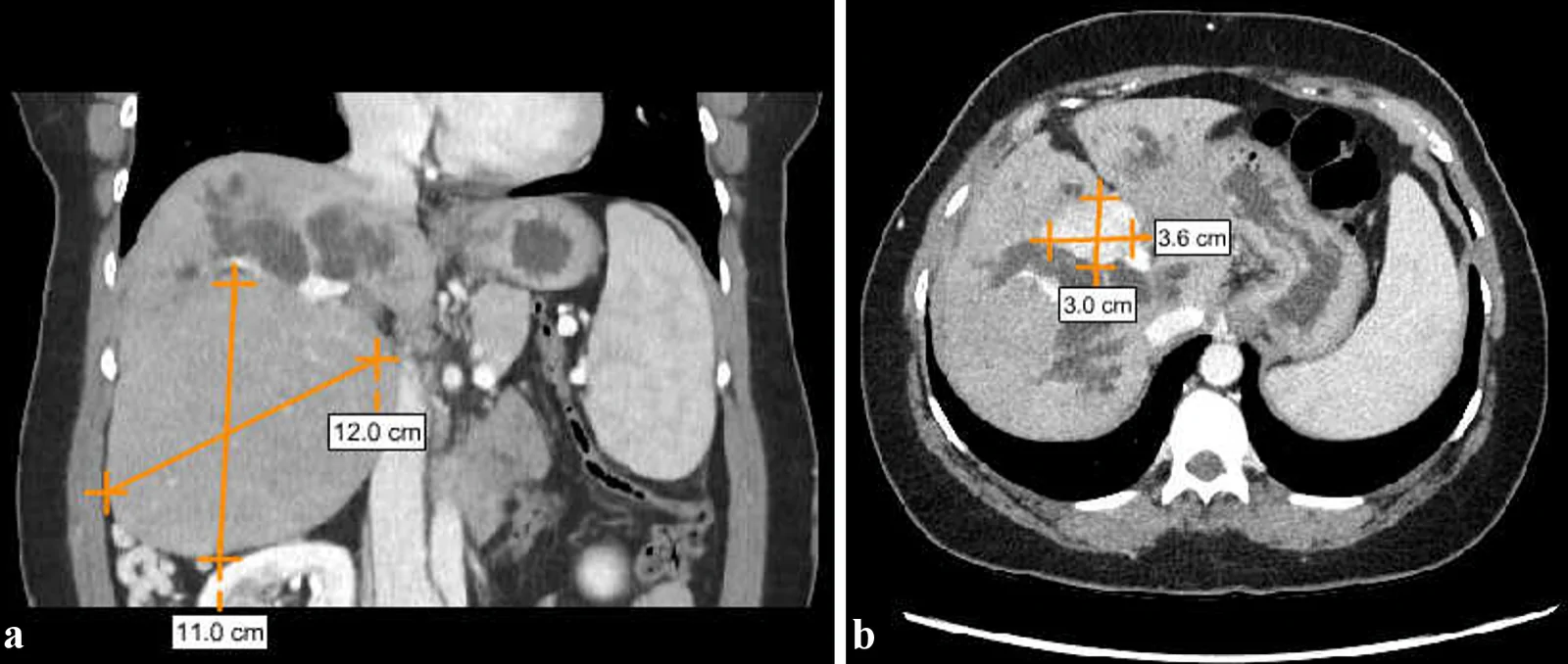
CT abdomen–pelvis with intravenous (IV) contrast enhancing, showing (a) markedly hypertrophied segment extending inferiorly from the liver (12 × 11 cm) and (b) a 3.6 × 3.0 cm tubular arterially enhancing structure anterior to the main portal vein. CT: computed tomography.

Computed tomography (CT) of the abdomen and pelvis with intravenous contrast demonstrated extensive chronic intrahepatic biliary ductal dilatation with associated hepatic parenchymal deformity. A markedly hypertrophied segment extending inferiorly from the liver (12 × 11 cm) corresponded to the previously identified mass-like lesion (Fig. 2a). At the hepatic hilum, a 3.6 × 3.0 cm tubular arterially enhancing structure anterior to the main portal vein, without internal parenchyma, was suspicious for arterial aneurysm or pseudoaneurysm (Fig. 2b). Additional findings included a small right porto-renal shunt and nonvisualization of the gallbladder despite no prior cholecystectomy. Further evaluation with MRI/magnetic resonance cholangiopancreatography (MRCP) and computed tomography angiography (CTA) was recommended.

The patient was subsequently evaluated by gastroenterology and hepatobiliary surgery. Because she remained asymptomatic with only mild bilirubin elevation, the abnormalities were assumed to be likely due to a chronic rather than an acute obstructive process. Differentials at this stage included congenital or autoimmune etiologies, as well as complex structural hepatobiliary disease.

**Figure 3:**
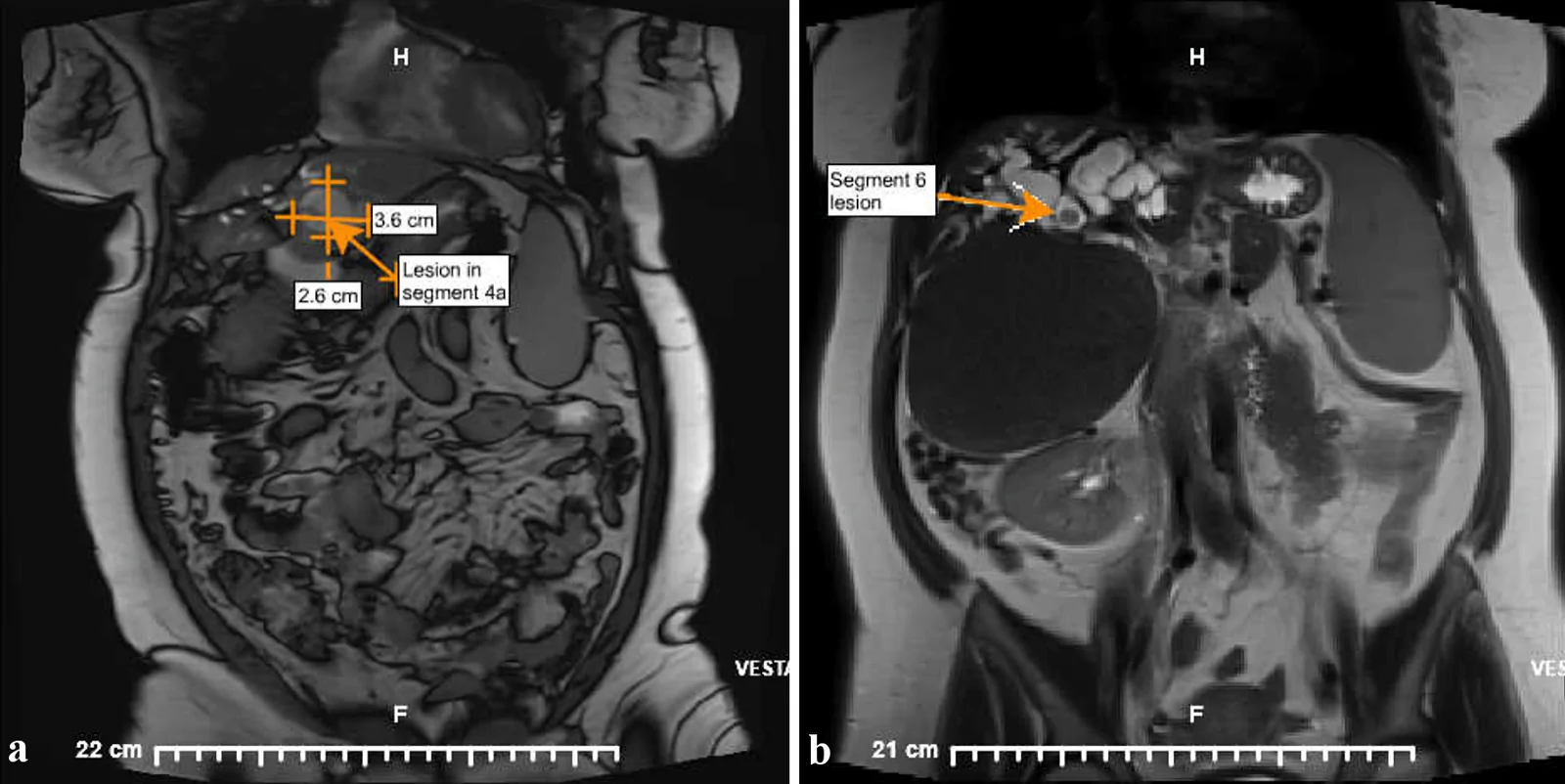
Magnetic resonance imaging with gadolinium of abdomen–pelvis, showing (a) an arterially enhancing lesion measuring 3.6 × 2.6 cm in segment 4a and (b) a second exophytic lesion arising from segment 6 which demonstrated different signal characteristics.

**Figure 4:**
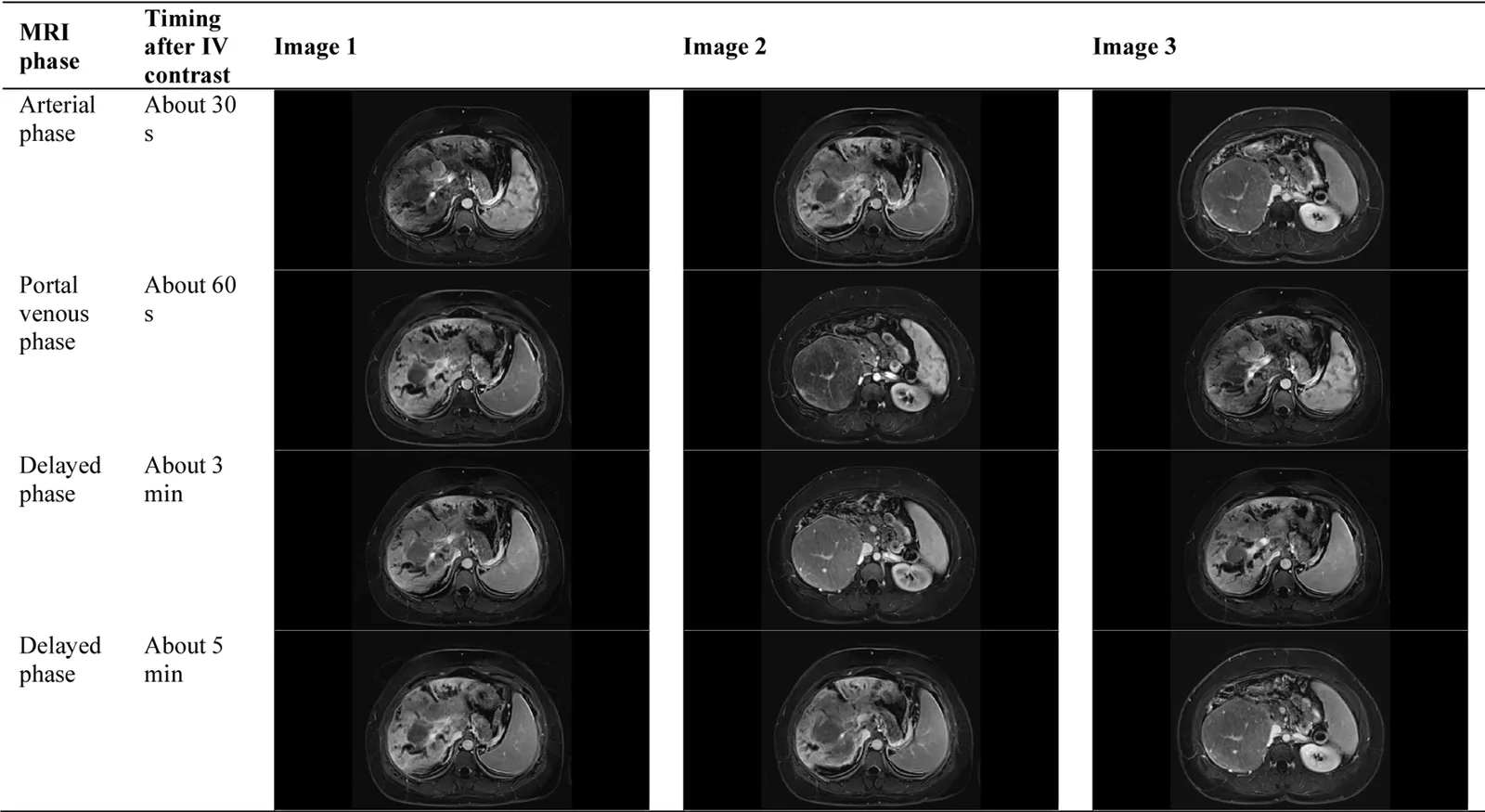
Triphasic Contrast-Enhanced MRI of the Liver Demonstrating Enhancement Characteristics of FNH With Patent Arterial/Venous Flow Through All Phases of Scan. MRI: magnetic resonance imaging; FNH: focal nodular hyperplasia; IV: intravenous.

MRI of the abdomen with gadolinium demonstrated significant intrahepatic biliary dilatation and two distinct hepatic lesions. The first was an arterially enhancing lesion measuring 3.6 × 2.6 cm in segment 4a that was relatively isointense to the liver on precontrast T1 imaging and on delayed sequences, with diffusion restriction, and was considered possibly consistent with atypical FNH (Fig. 3a). A second exophytic lesion arising from segment 6 demonstrated different signal characteristics, being T1 isointense, T2 hypointense, and hypointense relative to liver parenchyma on all post-contrast sequences, without diffusion restriction (Fig. 3b). This lesion was thought to be causing mass effect on the common hepatic duct with significant intrahepatic biliary dilatation. Its etiology remained uncertain, and atypical hepatocellular adenoma remained an important differential diagnosis because of its imaging characteristics and differing clinical implications [5]. The spleen measured 13 cm. The vascular status of the liver was unremarkable with patent flow through all contrast phases (Figure 4).

The case was discussed at a multidisciplinary hepatobiliary conference, where the overall appearance remained difficult to distinguish. Because of the complex anatomy, the marked biliary abnormalities, and subtle imaging findings suggestive of portal hypertension, including mild splenomegaly and a small porto-renal shunt, referral to a tertiary center with expertise in transplant surgery, portal hypertension, and congenital hepatobiliary disease was recommended.

**Figure 5:**
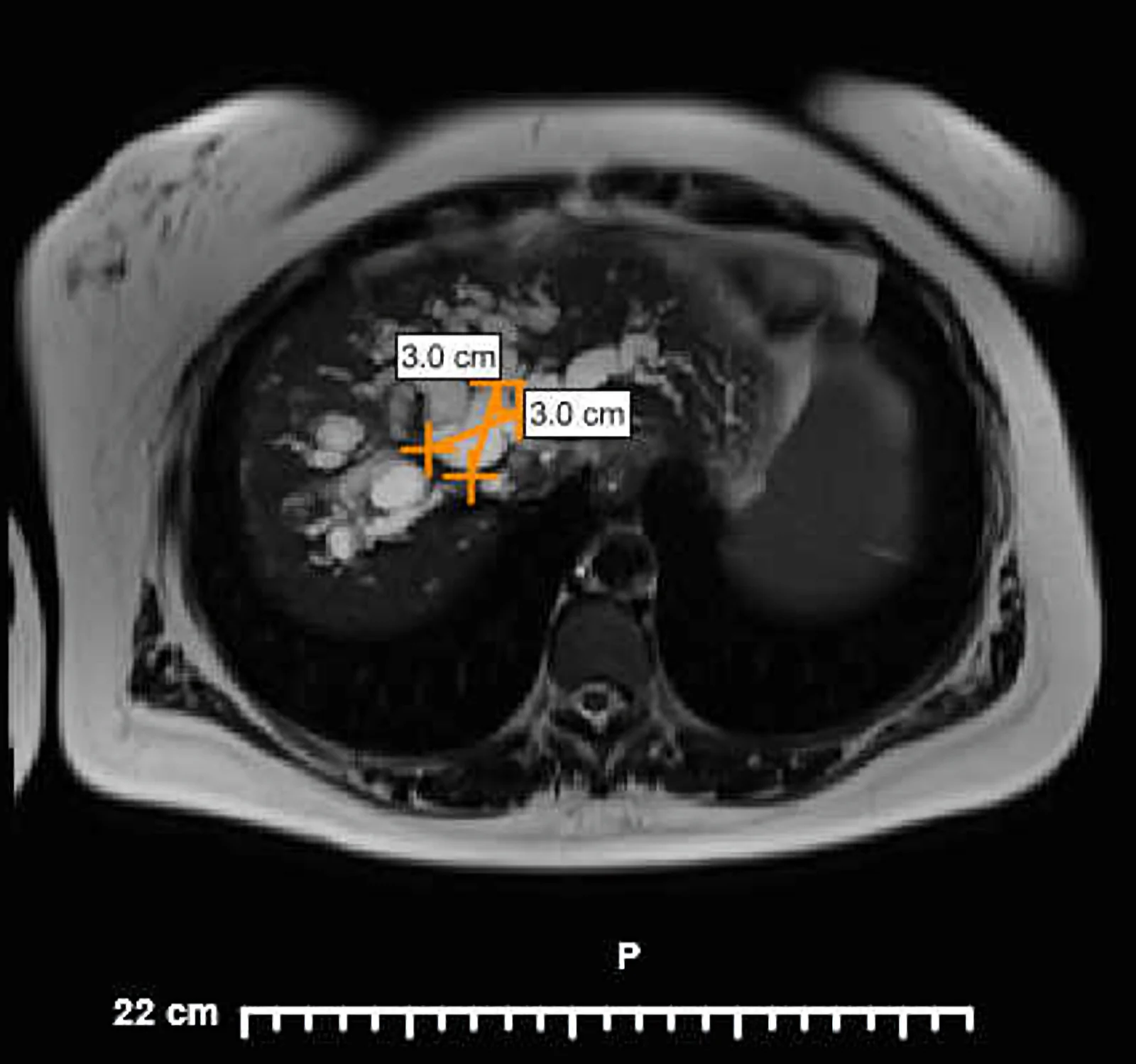
Magnetic resonance imaging with gadolinium of abdomen–pelvis, showing a lobulated arterially enhancing solid mass measuring 4.3 × 3.0 × 3.0 cm.

Follow-up MRI/MRCP 5 months later demonstrated persistent marked intrahepatic biliary ductal dilatation involving both the right and left intrahepatic ducts with compensatory hypertrophy of the inferior right hepatic lobe. At the confluence of the central hepatic ducts, there was a lobulated arterially enhancing solid mass measuring 4.3 × 3.0 × 3.0 cm that had not significantly changed in size (Fig. 5). There was an abrupt cutoff of the ducts at the level of the mass with convex bulging of the lesion into the duct, without evidence of extrinsic compression. The enhancement pattern was not consistent with arteriovenous malformation or hemangioma. The extrahepatic common bile duct was normal in caliber with normal tapering to the ampulla. There were no significant changes compared with the prior MRI study. The leading differential diagnosis in this study was intrahepatic bile duct adenoma, and endoscopic retrograde cholangiopancreatography (ERCP) was recommended, although it was not ultimately performed.

Serial imaging showed no significant progression, favoring a chronic process, but the nature of the lesion remained unclear. Repeat laboratory tests showed bilirubin levels between 1.2–1.5 mg/dL, AST levels between 56–76 U/L and AST levels between 46–59 U/L. Platelet levels remained normal throughout the entire presentation ranging from 125 to 183 × 10^3^/µL. This uncertainty became clinically relevant because the possibility of hepatic adenoma had implications for the patient’s plans to pursue pregnancy and the risk it carries, including concerns for lesion growth, rupture, or bleeding.

**Figure 6:**
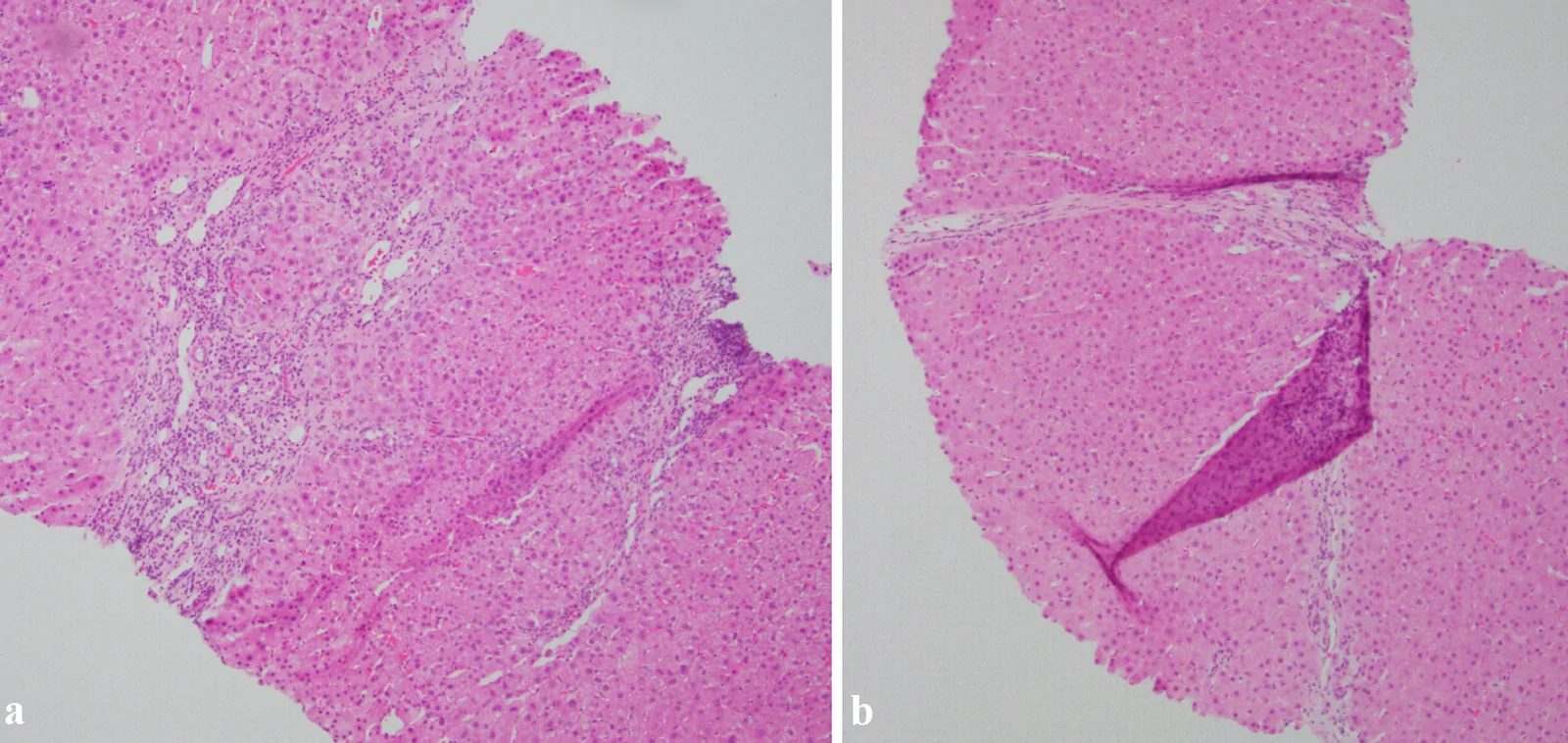
The two pictures, taken at × 10 magnification showing a nodule formed by a thin fibrous band. The fibrous septa also contain a bile ductular reaction and inflammatory cell infiltrates.

**Figure 7:**
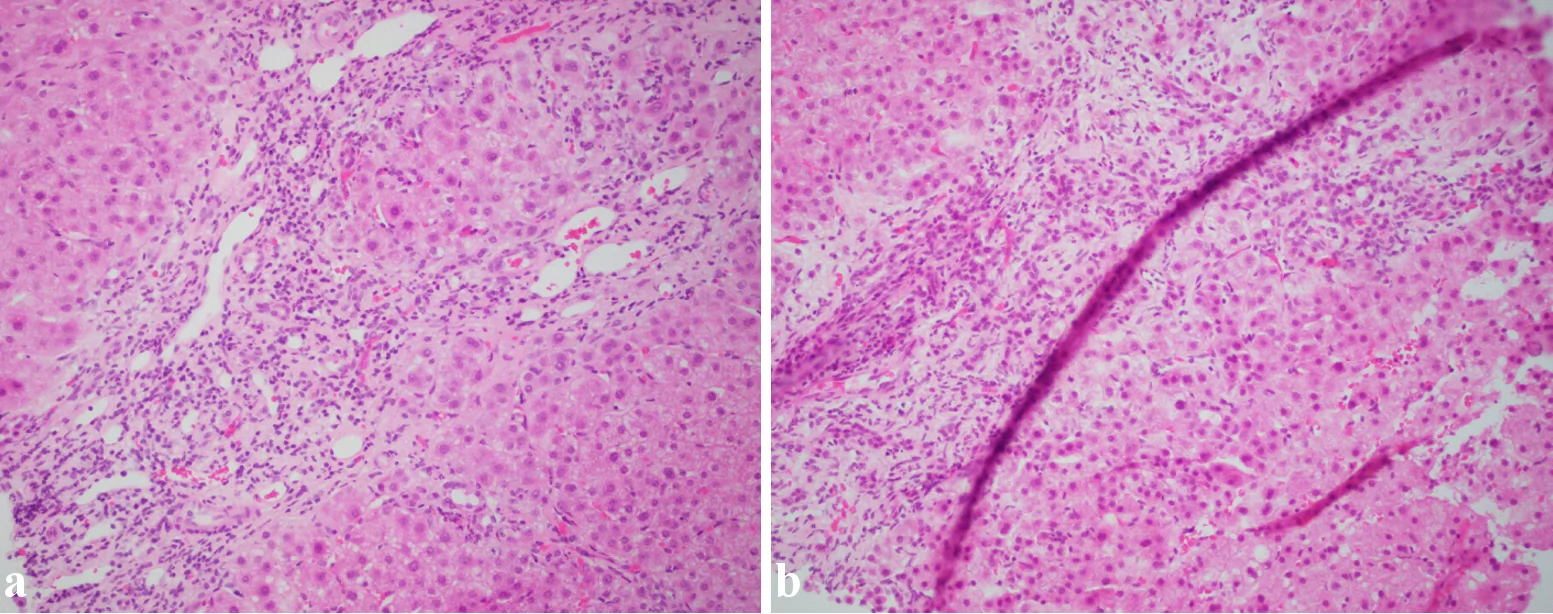
Two pictures showing (a) a fibrous band with abnormally dilated vessels and chronic inflammation and (b) a fibrous band with ductular reaction and chronic inflammation.

**Figure 8:**
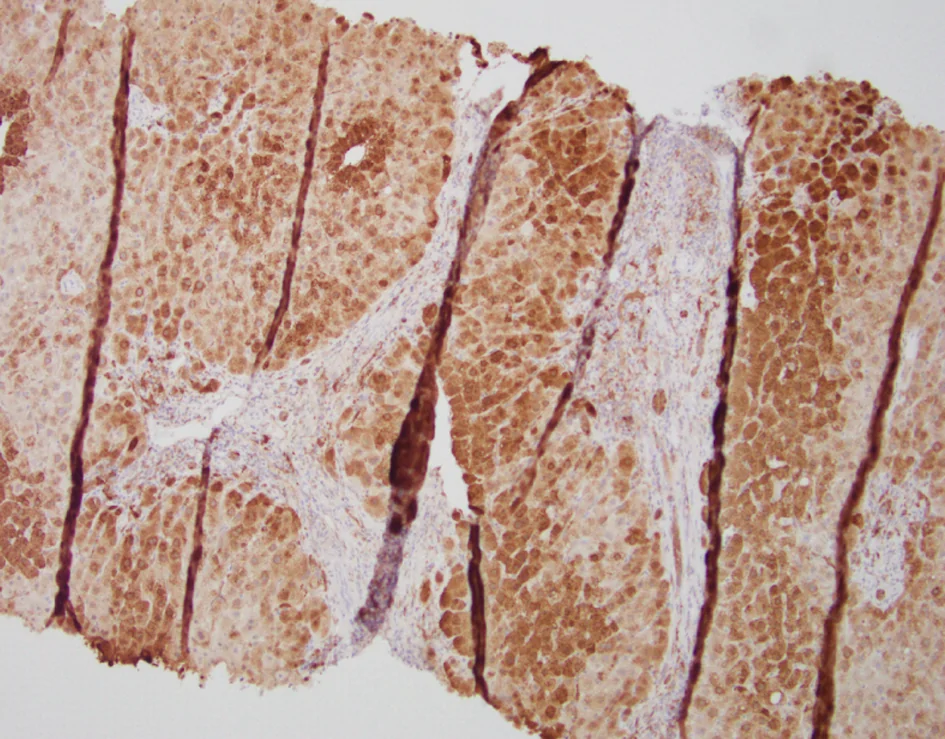
Glutamine synthetase immunostaining demonstrating a strong, patchy, map-like pattern.

Due to inconclusive results from imaging, a percutaneous liver biopsy was performed. Histopathologic examination demonstrated intermittent thin fibrous septa coursing across the liver lobules (Fig. 6). There was a focal area of sinusoidal dilatation and liver atrophy (Fig. 7a). Lobular architecture was otherwise preserved with distinct portal tracts and central veins. Some portal tracts were expanded by mild chronic inflammation and ductular reaction (Fig. 7b). There was no significant steatosis or ballooned hepatocytes, although a few hepatocytes with feathery degeneration were present. Trichrome stain highlighted areas of lobular fibrosis. Reticulin stain demonstrated preserved reticulin meshwork. Periodic acid-Schiff with diastase stain showed no alpha-1-antitrypsin globules, and the iron stain was negative. Immunohistochemistry showed map-like positivity for glutamine synthetase in hepatocytes and membranous staining for beta-catenin (Fig. 8), further supporting the diagnosis of FNH.

After biopsy, the lesion was determined to represent FNH, a benign lesion without malignant potential, but one that was causing chronic compression of the biliary tree. Suspicion for adenoma was effectively cleared, and the patient was informed that pregnancy was no longer contraindicated on this basis. Ongoing surveillance with repeat imaging and serial liver biochemical testing was recommended, and referral for tertiary hepatobiliary second opinion was advised because of the lesion’s unusual anatomy and chronic biliary involvement.

## Discussion

This case illustrates a rare and diagnostically challenging presentation of biopsy-proven FNH causing chronic intrahepatic biliary obstruction. FNH is generally asymptomatic, benign, and incidentally identified. In many cases, imaging is sufficiently characteristic to avoid biopsy. However, this patient’s presentation was highly atypical and created a broad and clinically meaningful differential diagnosis.

Several factors contributed to the difficulty of the diagnosis. First, the patient demonstrated persistent cholestatic-predominant liver biochemical abnormalities with elevated alkaline phosphatase, gamma-glutamyl transferase, and fluctuating bilirubin rather than normal liver tests typically seen with incidental benign lesions. Second, imaging showed marked intrahepatic biliary ductal dilatation, hepatic parenchymal deformity, apparent atrophy with compensatory hypertrophy, and a central enhancing structure initially interpreted as a possible aneurysm or pseudoaneurysm. Third, MRI suggested two lesions with differing signal characteristics, one favoring atypical FNH and another raising concern for atypical adenoma or another benign but indeterminate mass compressing the biliary tree. Fourth, mild splenomegaly and a porto-renal shunt suggested possible subtle portal hypertension, which broadened consideration to congenital or chronic hepatobiliary disorders.

The marked discordance between different imaging abnormalities and minimal symptoms added further complexity. Despite chronic biliary obstruction on imaging, the patient denied jaundice, pruritus, dark urine, pale stools, right upper quadrant pain, nausea, vomiting, and weight loss. This relative lack of symptoms argued against acute obstruction and suggested a slowly evolving process. Even so, the exact nature of the lesion remained unresolved on serial imaging alone.

The distinction between FNH and hepatocellular adenoma was particularly important. Hepatocellular adenoma may carry a risk of hemorrhage, interval growth, and, in some subtypes, malignant transformation [5]. In women contemplating pregnancy, this distinction may significantly affect counseling and management. FNH, by contrast, is generally benign and not considered premalignant. In this patient, concern for adenoma led to temporary caution regarding pregnancy until a definitive diagnosis was established.

Histopathologic examination was ultimately decisive. The biopsy showed preserved lobular architecture, intermittent thin fibrous septa, focal sinusoidal dilatation, mild chronic portal inflammation, ductular reaction, and preserved reticulin framework. Of particular importance, immunohistochemistry demonstrated map-like glutamine synthetase staining, a classic supportive finding in FNH, while beta-catenin showed membranous rather than nuclear staining. These findings, interpreted in conjunction with the imaging pattern and clinical course, supported the diagnosis of FNH and excluded adenoma.

Rare cases of symptomatic or compressive FNH have been described, including mass effect on adjacent structures, but biliary obstruction remains distinctly uncommon [7, 8]. In our case, the intrahepatic bile duct dilatation was attributed to chronic mass effect from the FNH lesion at the hepatic hilum, resulting in obstruction of the central biliary tree. Although congenital hepatobiliary abnormalities were considered early in the diagnostic evaluation because of the unusual imaging findings and chronic course, serial imaging, multidisciplinary review, and ultimately liver biopsy supported FNH as the underlying etiology. Therefore, congenital biliary disease was considered a differential diagnosis rather than the definitive cause of the biliary dilatation.

This case expands the clinical spectrum of FNH by demonstrating chronic intrahepatic biliary obstruction with long-standing cholestatic liver enzyme abnormalities and complex radiographic findings that mimicked vascular, neoplastic, and congenital hepatobiliary disorders. Although FNH is typically a benign and incidental liver lesion, it can rarely present with chronic biliary obstruction. When imaging findings are inconclusive, liver biopsy with immunohistochemistry may be essential to establish the diagnosis and guide appropriate management.

### Conclusions

FNH is usually an incidental benign liver lesion, but it may rarely present with chronic intrahepatic biliary obstruction and persistent cholestatic liver enzyme abnormalities. In this patient, atypical imaging features and mass effect on the biliary tree created prolonged diagnostic uncertainty and raised concern for adenoma, vascular lesion, intrahepatic bile duct adenoma, or congenital hepatobiliary disease. Definitive diagnosis required liver biopsy with immunohistochemistry. This case emphasizes the importance of integrating serial imaging, multidisciplinary review, and histopathology when hepatic lesions present atypically and when management decisions, including pregnancy counseling, depend on diagnostic certainty.

## Data Availability

The authors declare that data supporting the findings of this study are available within the article.

## Abbreviations

FNH: focal nodular hyperplasia
AST: aspartate aminotransferase
ALT: alanine aminotransferase
GGT: gamma-glutamyl transferase
MRI: magnetic resonance imaging
MRCP: magnetic resonance cholangiopancreatography
CT: computed tomography
CTA: computed tomography angiography
IV: intravenous
ERCP: endoscopic retrograde cholangiopancreatography
Ig: immunoglobulin
T1: T1-weighted imaging
T2: T2-weighted imaging
ACG: American College of Gastroenterology
